# Genome profiling of uropathogenic *E. coli* from strictly defined community-acquired UTI in paediatric patients: a multicentric study

**DOI:** 10.1186/s13756-023-01233-z

**Published:** 2023-04-18

**Authors:** Sarita Mohapatra, Dipannita Ghosh, Perumal Vivekanandan, Sneha Chunchanur, Shwetha Venugopal, Vibhor Tak, Rajashree Panigrahy, Susmita Chaudhuri, Swati Pundir, Tanya Sharma, Deepak Kocher, Harpreet Singh, Hitender Gautam, Seema Sood, Bimal Kumar Das, Arti Kapil, Arvind Kumar, Rajesh Kumari, Mani Kalaivani, Ambica Rangaiah, Harshal Ramesh Salve, Sumit Malhotra, Shashi Kant, Pankaj Hari, Investigators of CAUTION-ED STUDY (Community-acquired UTI & Emerging Drug Resistance), Sarita Mohapatra, K. C. Sneha, J. V. Shwetha, Vibhor Tak, Rajashree Panigrahy, Susmita Chaudhuri

**Affiliations:** 1 Department of Microbiology, AIIMS, New Delhi, India; 2 Kusuma School of Biological Sciences, Indian Institute of Technology Delhi, New Delhi, India; 3 Department of Microbiology, Bangalore Medical College and Research Institute, Bengaluru, India; 4 Department of Microbiology, AIIMS, Jodhpur, India; 5 Department of Microbiology, SUM Hospital, Bhubaneswar, India; 6 Translational Health Science and Technology Institute, Faridabad, India; 7 Department of Biomedical Informatics, ICMR, New Delhi, India; 8 Department of Medicine, AIIMS, New Delhi, India; 9 Department of Obstetrics and Gynaecology, AIIMS, New Delhi, India; 10 Department of Biostatistics, AIIMS, New Delhi, India; 11 Centre for Community Medicine, AIIMS, New Delhi, India; 12 Department of Paediatrics, AIIMS, New Delhi, India

**Keywords:** Urinary tract infection, Paediatric patient, Antimicrobial resistance, Community-acquired UTI, *E. coli*

## Abstract

**Background:**

Urinary tract infection (UTI) in children is a common bacterial infection. The emergence of extended-spectrum beta-lactamases (ESBLs) poses a major challenge against the treatment of uropathogens. We aimed to characterize the *E. coli* isolates recovered from children with UTI for their resistance profile and circulating sequence types (ST).

**Methods:**

Children (> 1.5–18 years of age) from different community health centres of India with symptoms of UTI were enrolled. Isolates causing significant bacteriuria were identified by Matrix-Assisted Laser Desorption Ionization Time of Flight Mass Spectrometry (MALDI-TOF MS) and tested for antimicrobial susceptibility by the automated system, VITEK-2 (Biomeriux, Durhum, US). Nineteen *E. coli* isolates (15 ESBL positive and 4 ESBL negative) were sequenced in Oxford Nanopore platform followed by core-genome phylogeny, accessory genome cluster analysis, identification of sequence types, mobile genetic elements, genetic antimicrobial resistance markers. The correlation between detection of antimicrobial resistance genes with phenotypic resistance profiles was also investigated.

**Results:**

Eleven percent of children had significant bacteriuria [male:female—1:1, > 50% were 11–18 years of age group]. *E. coli* was predominant (86%) followed by *K. pneumoniae* (11%). Susceptibility of *E. coli* was highest against fosfomycin (100%) followed by carbapenems (90.7%) and nitrofurantoin (88.8%). ST131 (15.8%) and ST167 (10.5%) found as high-risk clones with the presence of plasmid [IncFIB (63.1%), IncFIA (52.6%)], and composite transposon [Tn2680 (46.6%)] in many isolates. Few isolates coharboured multiple beta-lactamases including *bla*_NDM-5_ (33.3%), *bla*_OXA-1_ (53.3%), *bla*_CTX-M-15_ (60%) and *bla*_TEM-4_ (60%).

**Conclusions:**

This study highlights horizontal transmission of resistance genes and plasmids in paediatric patients at community centers across the nation harbouring multidrug-resistant genes such as *bla*_NDM-5_ and *bla*_CTX-M-15_ associated with high-risk clones ST131 and ST167. The data is alarming and emphasizes the need for rapid identification of resistance markers to reduce the spread in community. To our knowledge, this is the first multicentric study targeting paediatric UTI patients from the community setting of India.

## Background

Urinary tract infection (UTI) is a common occurrence in paediatric patients. Approximately, 8% of children experience at least one episode of UTI during some period of their childhood [1]. Lack of proper diagnosis and treatment may cause significant morbidity due to progressive destruction of the renal structure and may lead to chronic renal failure [2].

Global emergence of extended-spectrum beta-lactamases (ESBLs) producing uropathogens in community settings is of a great concern [3, 4]. Rapid dissemination of most of the beta-lactamases is facilitated by transferable plasmids that carry resistance genes to several other antibiotics [5]. Infections caused by ESBL-producing organisms are often difficult to treat because of the resistance to beta-lactams and coexisting resistance to other groups of drugs [6]. *Escherichia coli* among the Enterobacterales is the major uropathogen in the paediatric age group reported from both community and hospital settings [7–9]. *E. coli* producing ESBLs is a global concern due to limited available therapeutic options. There is a rising trend reported in the occurrence of community-acquired UTI from the western world caused by ESBL-producing *E. coli* among the paediatric population [2, 10]. However, there is scarce knowledge about the prevalence, and molecular epidemiology of resistance genes associated with paediatric UTI in the community setting, especially in the South Asian region including Indian subcontinent [11, 12]. This study reports the draft genome sequence and analysis of 19 uropathogenic *E. coli* (UPEC) (15 ESBL positive and 4 ESBL negative) isolates from paediatric patients with strictly defined community-acquired UTI from four different geographical locations of India. The study also aimed to determine the distribution of antimicrobial resistance (AMR) genes and the plasmid replicons that help in their replication. This information would be helpful in understanding the epidemiology and development of control and prevention strategies in community settings of developing nations like India.

## Methods

This was a prospective multicentric cross-sectional study conducted at the Community Health Centers (CHC) from four different geographical regions of India over a period of three years. It was funded by Indian Council of Medical research in 2019 and conducted by the CAUTION-ED study group. Ethical clearance for the study was obtained by the nodal center (IEC-192/05.04.2019, RP-28/2019). Informed consent for these patients was obtained from their guardians. Consecutive patients with age > 1.5 years to 18 years from the defined community health centers presenting to OPD with increased frequency, urgency, burning, or unexplained fever as chief complaints were included in this study. Patients with vesicoureteral reflex and on history of antibiotic prophylaxis were excluded from the study. Clean catch mid-stream urine sample was collected for processing. Growth of single organism with colony count ≥ 10^3^ (CFU/ml) was considered significant bacteriuria. Identification of isolates to species level was done using Matrix-Assisted Laser Desorption Ionization Time of Flight Mass Spectrometry (MALDI-TOF MS) (Biomeriux, Germany) and Antibiotic Susceptibility Testing (AST) was performed by VITEK-2 (Biomeriux, Durhum, US). The different cards used for antimicrobial susceptibility testing by VITEK -2 were GN (N-235) for Lactose Fermenting (LF) Bacteria, GN (N-281) for Non Lactose Fermenting (NLF) Bacteria and GP (N-628) for Gram positive Bacteria (GPC). MIC_50_ and MIC_90_ for different antimicrobial agents were also calculated.

### Whole genome sequencing, assembly and annotation

Nineteen UPEC isolates (15 ESBL + ve and 4 ESBL − ve) from the total 54 UPEC isolates were randomly selected for whole genome sequencing (WGS) from all four different geographical regions. WGS was performed to determine the molecular distribution of AMR genes, transposable genetic elements like plasmids, transposons and replicons and their relationship with sequence types.

Genomic DNA from 19 UPEC isolates were extracted using QIAamp DNA Mini Kit (Qiagen, Germany) using manufacturer’s protocol. Library for Oxford Nanopore sequencing were prepared using the PCR-free Native Barcode Expansion kit (EXP-NBD104) and Ligation Sequencing kit (SQK-LSK109). Reads with Q score ≥ 8 were used to find antibiotic resistance markers in ARMA (antimicrobial resistance mapping application) workflow from the EPI2ME analysis platform (https://epi2me.nanoporetech.com). The reads were also used to find plasmids in PlasmidFinder 2.0- https://cge.food.dtu.dk/services/PlasmidFinder/ (accession date: 22/11/21) [13, 14]. Assemblies were made using Minimap2 [15] and NC_000913.3 *E. coli* str. K-12 substr. MG1655 was used as reference. Samtools and Bcftools [16] were used to find alignment statistics, perform variant calling and obtain consensus sequences. Chromosomal mutations associated with antibiotic resistance were assessed using ResFinder 4.1- https://cge.food.dtu.dk/services/ResFinder/ (accession date: 16/11/21) [17]. The assemblies were annotated using Prokka [18] and pan-genome analysis was performed with Roary [19]. Accessory genome presence/absence was used for hierarchical clustering in Pvclust [20] with 1000 bootstrap iterations. The assemblies were also analysed for the identification of Multi Locus Sequence types (MLST) in MLST 2.0- https://cge.food.dtu.dk/services/MLST/ (accession date: 18/11/21) [21–23]. The mobile genomic elements were predicted using Mobile Element Finder-https://cge.food.dtu.dk/services/MobileElementFinder/ (accession date: 19/07/2022).

### Phylogenetic analysis of core genomes

Snippy-core [24] was used to perform core-genome alignment for all 19 isolates. A rooted Maximum Likelihood phylogenetic tree was created using MEGA11 [25] with 1000 bootstrap iterations. NC_011740.1 *E. fergusonii* ATCC 35,469 was used as an outgroup. Annotation of the tree with MLST and plasmids was done in Interactive Tree Of Life (iTOL) [26].

## Results

### Demographic features

A total of 658 urine specimens were tested following standard operative protocol during the study period. More than fifty percent of patients belonged to age group of 11–18 years (55%), followed by a similar number in both 6–10 years and 0–5 years age groups (22.4%) with equal affection of male and female. Total of 73 uropathogens (11%) were isolated causing significant bacteriuria confirming UTI. *E. coli* species was observed as the most prominent with a prevalence rate of 86% (n = 54), followed by *Klebsiella pneumoniae* (n = 7, 11%), *Proteus* spp. (n = 2, 3%) *Acinetobacter* spp. (n = 2, 2.7%), *Pseudomonas aeruginosa* (n = 1, 1.4%), *Enterococcus* spp. (n = 6, 8.2%), and *Staphylococcus aureus* (n = 1, 1.3%). 63% (34/54) of *E. coli* isolates were found ESBL-positive. Among the *E. coli* isolates, the highest resistance was observed against ticarcillin (74%), followed by ampicillin (72.3%), cefalothin (70.4%), ciprofloxacin (68.6%), cefixime (63%) and ceftriaxone (61.2%). More than one-third isolates were resistant to amoxicillin-clavulanic acid (50%), piperacillin-tazobactam (50%), ceftazidime (48.2%), cotrimoxazole (47%), cefoxitin (37%) and gentamicin (32.5%). Approximately, 20% of isolates were found resistant to amikacin (20.4%). The resistance against ertapenem and nitrofurantoin was observed as 9.3% and 12.2%. All the isolates revealed 100% susceptibility against fosfomycin. MIC_50_ for *E. coli* isolates against ceftriaxone was observed 16 times that of its resistance breakpoint. (Table 1).

**Table 1:** Antimicrobial susceptibility of major uropathogens causing community-acquired urinary tract among the paediatric patients

Antibiotics	MIC Break points + (S, R)*	*E. coli (54)*				*K. pneumoniae (7)*			
		S = n (%)	MIC50_50_	MIC_90_	MIC Range	S = n (%)	MIC_50_	MIC_90_	MIC Range
Amikacin	S ≤ 16, R ≥ 64	43 (79.6%)	2	64	≤ 2 to ≥ 64	7 (100%)	2	32	≤ 2 to ≥ 64
Amoxicillin-clavulanic acid	S ≤ 8/4, R ≥ 32/16	27 (50%)	16	32	≤ 2 to ≥ 32	5 (71.4%)	8	–	≤ 2 to ≥ 32
Ampicillin	S ≤ 8, R ≥ 32	15 (27.7%)	32	32	≤ 2 to ≥ 32	1 (14.2%)	32	32	≤ 2 to ≥ 32
Cefalotin	S ≤ 16, R ≥ 32	16 (29.6%)	64	64	≤ 2 to ≥ 64	4 (57%)	4	–	≤ 2 to ≥ 64
Cefixime	S ≤ 1, R ≥ 4	20 (37%)	4	4	≤ 0.25 to ≥ 4	7 (100%)	0.25	4	≤ 0.25 to ≥ 4
Cefoxitin	S ≤ 8, R ≥ 32	34 (63%)	4	64	≤ 2 to ≥ 64	4 (57%)	4	–	≤ 4 to ≥ 64
Ceftazidime	S ≤ 4, R ≥ 16	28 (51.8%)	4	64	≤ 1 to ≥ 64	3 (42.8%)	1	64	≤ 0.5 to ≥ 64
Ceftriaxone	S ≤ 1, R ≥ 4	21 (38.8%)	64	64	≤ 1 to ≥ 64	6 (85.7)	1	64	≤ 1 to ≥ 64
Ciprofloxacin	S ≤ 0.25, R ≥ 1	17 (31.4%)	4	4	≤ 0.25 to ≥ 64	3 (42.8%)	0.25	4	≤ 0.25 to ≥ 4
Cotrimoxazole	S ≤ 40, R ≥ 80	29 (53.7%)	20	320	≤ 20 to ≥ 320	5 (71.4%)	20	320	≤ 20 to ≥ 320
Ertapenem	S ≤ 0.5, R ≥ 2	49 (90.7%)	0.5	0.5	≤ 0.5 to ≥ 8	7 (100%)	0.5	–	≤ 0.5 to ≥ 8
Fosfomycin	S ≤ 64, R ≥ 256	54 (100%)	16	16	< = 16 to ≥ 128	–	–	–	
Gentamicin	S ≤ 4, R ≥ 16	37 (68.5%)	1	16	≤ 1 to ≥ 16	6 (85.7%)	1	8	≤ 1 to ≥ 16
Nitrofurantoin	S ≤ 32, R ≥ 128	48 (88.8%)	16	64	≤ 16 to ≥ 256	5 (71.4%)	64	128	≤ 16 to ≥ 512
Piperacillin/Tazobactam	S ≤ 32, R > 128	27 (50%)	64	128	≤ 4 to ≥ 128	6 (85.7%)	4	128	≤ 4 to ≥ 128
Ticarcillin	S ≤ 8, R ≥ 32	14 (26%)	128	128	≤ 8 to ≥ 128	0%	128	128	≤ 8 to ≥ 128

^*^S: Susceptible, R: Resistant; + as per CLSI M100 document, MIC_50_/MIC_90_ of individual drugs refers to the minimum inhibitory concentration of antibiotics that inhibits the growth of 50% or 90% isolates, respectively

### Genomic features

**Fig. 1:**
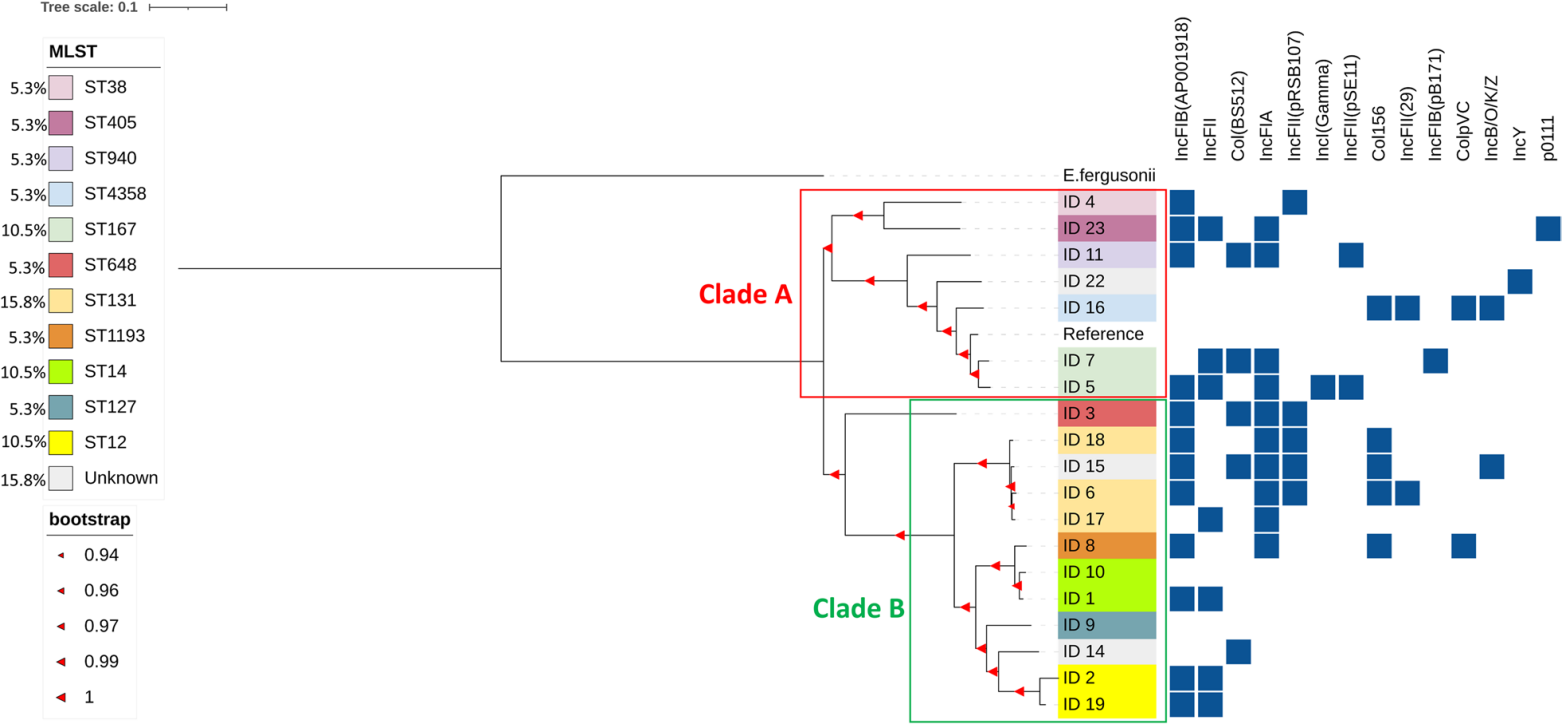
Phylogenetic analysis of core genome of *Escherichia coli* isolates from paediatric patients. A rooted maximum likelihood phylogenetic tree constructed using SNPs across the core-genomes of the 19 *E. coli* isolates, reference (NC_000913.3) and outgroup (*E. fergusonii*, NC_011740.1). The phylogram has been annotated with the MLST types of the 19 isolates as well as the plasmids identified in each of them. Number of bootstrap iterations: 1000

All nineteen isolates (15 ESBL + ve and 4 ESBL − ve) were identified as *E. coli* by EPI2ME analysis platform from Oxford Nanopore Technologies. Genome assembly was done by mapping the sequencing reads with Q score ≥ 8 to *E. coli* MG1655 (Accession number: NC_000913.3) as described in the methods section. The chromosome lengths of the assembled genomes for each of the 19 strains ranged from 4.67 to 4.8 Mbp. MLST analysis revealed that 16 isolates belonged to 11 different MLST types while the remaining three did not match any known Sequence Type (ST) (Fig. 1). Plasmid identification from raw reads showed several isolates harboured multiple plasmids that carry antibiotic resistance genes (ARGs) and they have been annotated in Fig. 1. ST131 was the most prevalent phylogroup identified among the isolates. Plasmid IncFIB was found to be present in 63.1% (12/19) followed by IncFIA that was detected among 52.6% (10/19) of the sequenced isolates. Hierarchical cluster analysis of all the 19 *E. coli* isolates was performed after constructing an accessory genome presence–absence matrix.

### Phylogenetic and cluster analysis

**Fig. 2:**
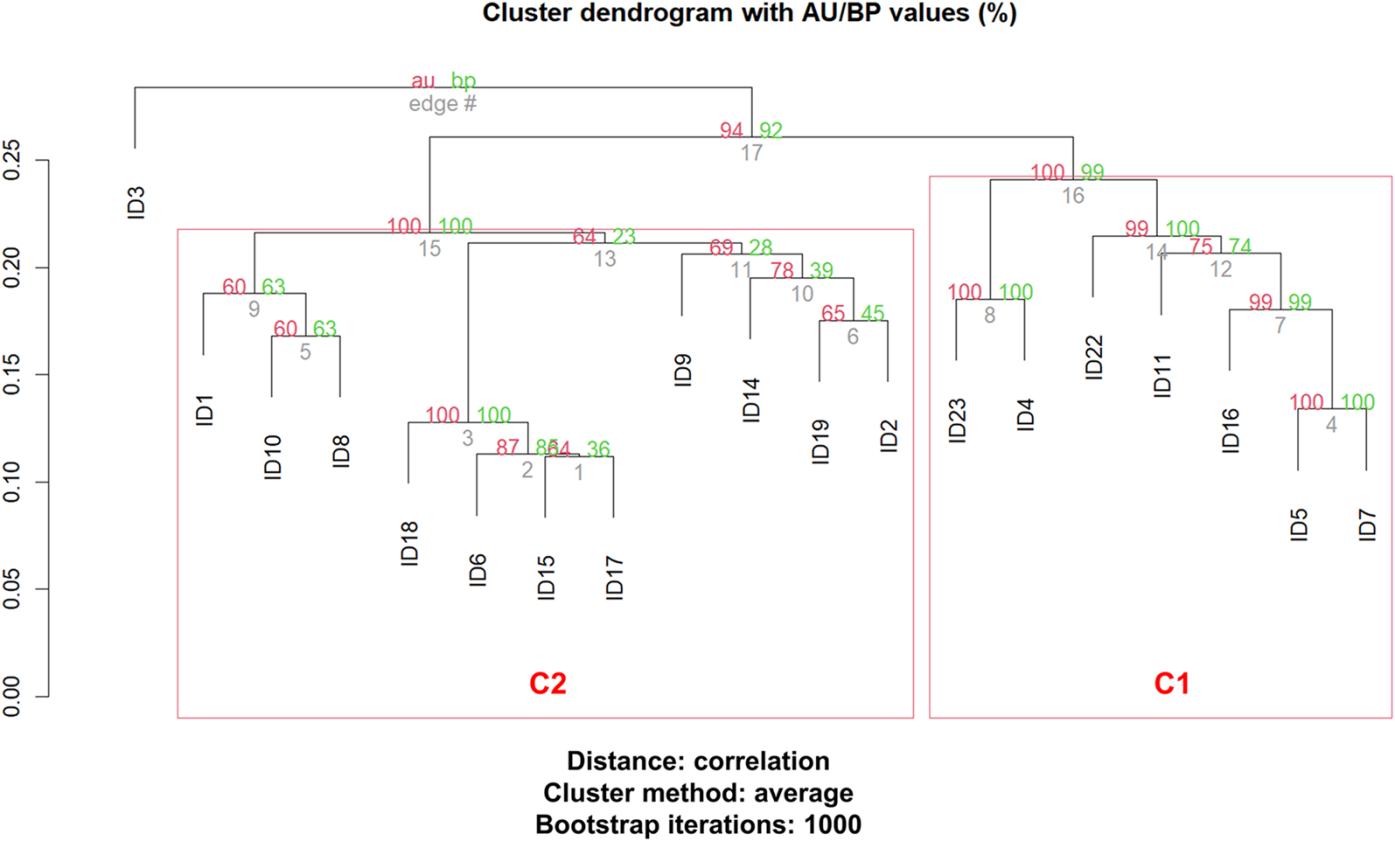
Hierarchical cluster dendrogram based on the accessory genome. The dendrogram branches into two major clusters, labelled as C1 and C2, in red. The probability value for significant clusters is ≥ 0.95

Core genome alignment of the 19 *E. coli* isolates and the reference genome (*E. coli* MG1655, Accession number: NC_000913.3) was performed using Snippy-core (https://github.com/tseemann/snippy) to identify a total of 51,314 single nucleotide polymorphisms (SNPs) between them. A rooted phylogenetic tree was constructed using *E. fergusonii* ATCC 35,469 (Accession number: NC_011740.1) as an outgroup (Fig. 1). Two distinct clades were observed in the phylogenetic tree (Clade A and Clade B), which further branched into subclades. Based on hierarchical cluster analysis, the 19 *E. coli* isolates were observed in two distinct clusters, C1 and C2 (Fig. 2). It was observed that all the isolates from clade A constitute the core-genome phylogeny cluster together in cluster C1 and the isolates from clade B form cluster C2. Isolates ID6, ID17 and ID18 of cluster B2 were further branched into a subclade in clade B and were identified to be ST131. ID15 being part of the same subclade was not identified as ST131. Six ESBL-positive isolates from Clade B were observed to carry lncFIAplasmid. Isolates ID1, ID10 and ID8 formed a separate subclade of which ID1 and ID10 belonged to ST14 and ID8 belonged to ST1193. Similarly, isolates ID19 and ID2 from the same subclade branching from Clade B belonged to ST12 carrying plasmids lncFIB and lncFII. In Clade A, a similar sub-branching was observed in cluster C1 for isolates ID23 and ID4. Both of these isolates were found to carry plasmid lncFIB. Isolates ID5 and ID7 from Clade A were identified as ST167 and clustered together in cluster C1 of the dendrogram.

### Correlation of genetic antibiotic resistance markers to phenotype

Genetic antibiotic resistance profiling of all 19 isolates using EPI2ME ARMA workflow [CARD database [27]] and ResFinder 4.1 [28] showed multiple drug resistance genetic markers against β-lactams, aminoglycosides, sulphonamides, quinolones, macrolide, and co-trimoxazole (Table 2). All fifteen isolates phenotypically identified as ESBL producers harbour at least one of the β-lactamase genes, including *bla*_TEM_ (− 4, − 1, − 76, − 33, − 166), *bla*_CTX-M_ (− 15, − 101, − 55, − 33), *bla*_OXA_ (− 1, − 320, − 224), *bla*_ACT_ (− 5, − 14), and *bla*_NDM_ (− 5, − 4, − 9). The majority of isolates were observed to carry *bla*_CTX_ and *bla*_TEM_ types of ESBL genes. *bla*_CTX-M-15_ gene was detected in 60% (9/15) followed by *bla*_CTX-M-55_ (53.3%, 8/15) and *bla*_CTX-M-33_ (53.3%, 8/15). Among *bla*_TEM_ genes, *bla*_TEM-4_was the most prevalent present in 60% (9/15) of isolates. Among the carbapenemases, 5 isolate were found to harbour *bla*_NDM-5_ followed by 3 isolates with *bla*_NDM-4_. *bla*_OXA_ gene was detected in 53.3% (8/15) of the isolates, with *bla*_OXA-1_ being the predominant one. *bla*_ACT-5_ was found in 17 isolates followed by *bla*_ACT-14_. Chromosomal point mutations in the genes associated with quinolone resistance was observed for *gyrA* (n = 17, 89%), *parC* (n = 12, 63%) and *parE* (n = 10, 53%). Plasmid-mediated resistance markers for quinolone were present in 3 of the isolates, ID2 (*qnrS1*, *qnrS3* and *qnrS4*), ID14 (*qnrS1*) and ID19 (*qnrB4*). Twelve isolates (63.2%) carried resistance genes for trimethoprim including *dfrA1*, *dfrA12*, *dfrA14* and *dfrA17*. Genetic markers for aminoglycoside resistance including aminoglycoside phosphotransferases (APHs) (n = 10, 53%), aminoglycoside adenylyltransferases (*aadA*) (n = 11, 58%) and aminoglycoside acetyltransferases (AAC) (n = 6, 32%) were observed in several isolates. Most of the isolates from Clade A were observed to carry multiple aminoglycoside adenylyltransferases whereas isolates from Clade B were observed to carry multiple aminoglycoside acetyltransferases genes. APH(6)-Id was the most prevalent phosphotransferase followed by APH(3ʺ)-Ib and APH(3ʹ)-IIa. Macrolide resistance gene *mphA* was found to be present in 47.3% of all the isolates sequenced in the study. However, we did observe few discordant results between the phenotype and genotype for example: isolate 22 and 23 found ESBL-negative despite of presence of beta lactamase genes *bla*_CTX-M-15_ and *bla*_TEM_. Similar discordant observation were also found incase of cefoxitin susceptibility in isolate no 7, 16, 1, 2 ,6, 9, 10. These discordant observations might be explained by the possibility that mere presence of the resistance genes does not always warrant expression and phenotypic exhibition.

**Table 2:** Phenotypic resistance profiles with corresponding genotypic resistance markers of the *Escherichia coli* isolates

Isolate	Clade	ESBL	Antibiotic resistance profile	Chromosomal Point Mutations	Beta-lactamases			Aminogylcosides	Quinolones resistance	Sulfonamide resistance	Trimethoprim resistance	Macrolide resistance
					ESBL	AmpC	Carbapenem					
					TEM, CTX-M	ACT	OXA, NDM		Qnr	Sul	dfrA	mpH
4	A	Positive	Amoxicillin/clavulanic acid, Ampicillin, Cefalotin, Cefixime, Cefoxitin, Ceftazidime, Ceftriaxone, Ciprofloxacin, Co-trimoxazole, Gentamicin, Piperacillin/tazobactam, Ticarcillin	*gyrA*: S83L, D87N *parC*: S57T, S80I *parE*: S458A	TEM (4,166) CTX-M (42, 27, 3, 55, 142, 15, 33, 88, 66)		OXA-1 NDM-5	APH(6)-Id aadA (2, 3, 2)		Sul (2,1)	(dfrA1, 14, 12)	mphA
5	A	Positive	Amoxicillin/clavulanic acid, Ampicillin, Cefalotin, Cefixime, Cefoxitin, Ceftazidime, Ceftriaxone, Ciprofloxacin, Co-trimoxazole, Gentamicin, Nitrofurantoin, Norfloxacin, Piperacillin/tazobactam, Ticarcillin, Ertapenem	*gyrA*: S83L, D87N *parC*: S80I *parE*: S458A	TEM- (4, 33)	ACT-5	NDM (5, 4, 2, 7, 1, 9)	aadA (2, 3, 17)		sul 1	dfrA12	mphA
7	A	Positive	Amoxicillin/clavulanic acid, Ampicillin, Cefalotin, Cefixime, Ciprofloxacin, Co-trimoxazole, Ticarcillin, Ertapenem	*gyrA*: S83L, D87N *parC*: S80I *parE*: S458A	TEM- (4, 159, 166,1)	ACT- (5, 14)	OXA-1 NDM (5,4,8)	aadA2			dfrA12	
11	A	Negative	Ampicillin, Co-trimoxazole	*gyrA*: S83L, D87N *parC*: S80I *parE*: S458A	TEM- (4,158, 33, 148, 1, 169) CTX-M (15, 117, 103, 3, 55, 82, 52, 101, 114, 88, 66, 42, 28, 79, 71)			AadA (8, 23, 24)			dfrA1	mphA
16	A	Positive	Amoxicillin/clavulanic acid, Ampicillin, Cefalotin, Cefixime, Ceftazidime, Ceftriaxone, Ciprofloxacin, Norfloxacin, Piperacillin/tazobactam, Ticarcillin	*gyrA*: S83L	TEM (4, 166)	ACT- (5, 14)	OXA-1	APH ((6)-Id, (3ʺ)-Ib)		Sul (1, 2)	dfrA17	mphA
22	A	Negative	Cefalotin			ACT-5						
23	A	Positive	Ampicillin, Cefalotin, Cefixime, Cefoxitin, Ceftazidime, Ceftriaxone, Ciprofloxacin, Co-trimoxazole, Gentamicin, Norfloxacin, Piperacillin, Ticarcillin	*gyrA*: S83L, D87N *parC*: S80I *parE*: S458A	CTX-M (15,82,139,54,114,80,22,224,33,117,71)	ACT-5	OXA- (1, 320)	AAC(3)- (IIc, IIa) aadA5		Sul1	dfrA17	
1	B	Positive	Ciprofloxacin	*gyrA*: S83L		ACT- (14, 5)		APH(6)-Id, (3ʺ)-Ib)		sul2		
2	B	Positive	Ampicillin, Cefalotin, Cefixime, Ceftriaxone, Ciprofloxacin, Ticarcillin	*gyrA*: S83L, D87N *parC*: E84K	TEM- (4, 76, 95,1,71, 115) CTX-M (15, 55, 101, 114, 82, 33, 103, 22, 132, 3, 61)	ACT- (5, 14)			Qnr (S1, S3, S4)	Sul (2,1)		
3	B	Positive	Amoxicillin/clavulanic acid, Ampicillin, Cefalotin, Cefixime, Cefoxitin, Ceftazidime, Ceftriaxone, Ciprofloxacin, Co-trimoxazole, Ertapenem, Gentamicin, Piperacillin/tazobactam, Ticarcillin	*gyrA*: S83L, D87N *parC*: S80I	TEM- (4,1, 206, 33, 166, 198,143) CTX-M (55, 15, 114, 33, 52, 101, 66, 79, 71, 142)	ACT-(5, 14)	OXA-1 NDM- (5, 4, 9)	AAC(3)-IIc (3)-IIa, (6')-Ib)		sul1	dfr (A17, A12)	mphA
6	B	Positive	Ampicillin, Ceftriaxone, Ciprofloxacin	*gyrA*: S83L, D87N *parC*: S80I, E84V *parE*: I529L	CTX-M (27, 129, 102, 85, 9, 19, 98, 47, 113, 48)	ACT-5		APH(6)-Id, (3ʺ)-Ib aadA5		Sul (1, 2)	dfrA17	mphA
8	B	Positive	Ampicillin, Cefalotin, Cefixime, Cefoxitin, Ceftazidime, Ceftriaxone, Co-trimoxazole, Ticarcillin	*gyrA*: S83L, D87N *parC*: S80I *parE*: L416F	TEM- (4,76, 70, 166) CTX-M (15, 55, 117, 71, 103, 82, 139, 101, 69,3, 54, 114, 22, 79, 33, 88)	ACT-(5, 14)		APH(6)-Id aadA5		Sul (1, 2)	dfrA17	mphA
9	B	Positive	Ampicillin, Cefalotin, Cefixime, Ceftriaxone, Ciprofloxacin, Co-trimoxazole, Ticarcillin	*gyrA*: D87G	CTX-M (15, 82, 55, 114, 71, 3, 117, 139, 54, 33, 88, 142, 103, 123, 79)	ACT-(5, 14)						
10	B	Negative	Ciprofloxacin	*gyrA*: S83L		ACT-(5,14, 7)		APH(3ʹ)-IIa				
14	B	Positive	Amoxicillin/clavulanic acid, Ampicillin, Cefalotin, Cefixime, Cefoxitin, Ceftazidime, Ceftriaxone, Ciprofloxacin, Ertapenem, Norfloxacin Piperacillin/tazobactam, Ticarcillin			ACT-(5,14, 9)			QnrS1	sul2	dfrA12	
15	B	Positive	Amoxicillin/clavulanic acid, Ampicillin, Cefalotin, Cefixime, Ceftazidime, Ceftriaxone, Ciprofloxacin, Gentamicin, Norfloxacin, Piperacillin, Ticarcillin	*gyrA*: S83L, D87N *parC*: S80I, E84V *parE*: I529L	TEM- (4,176, 105, 163) CTX-M (15,82,101,103,3,62,55,33,71,142)	ACT-(5,14)	OXA-1 NDM-5	APH(6)-Id, (3ʺ)-Ib) AAC ((3)-IIc, (3)-Iia)		sul2		
17	B	Positive	Ampicillin, Cefalotin, Cefixime, Ceftazidime, Ceftriaxone, Ciprofloxacin, Gentamicin, Norfloxacin, Ticarcillin	*gyrA*: S83L, D87N *parC*: S80I, E84V *parE*: I529L	TEM- (4,176, 76, 148, 146, 137) CTX-M (15,117,55,82,22,132,28,53,71,88,72)	ACT-5	OXA-1	AAC(3)-IIc, (3)-IIa)				
18	B	Positive	Amoxicillin/clavulanic acid, Ampicillin, Cefalotin, Cefixime, Ceftazidime, Ceftriaxone, Ciprofloxacin, Co-trimoxazole, Gentamicin, Norfloxacin, Piperacillin, Ticarcillin	*gyrA*: S83L, D87N *parC*: S80I, E84V *parE*: I529L	CTX-M (15,42,114,82,123,55,79,33,117)	ACT- (5, 14)	OXA-1	APH(6)-Id, (3ʺ)-Ib), aadA5		Sul (1, 2)	dfrA17	mphA
19	B	Negative	Amoxicillin/clavulanic acid, Ampicillin, Cefalotin, Cefixime, Cefoxitin, Ceftazidime, Ceftriaxone, Ciprofloxacin, Co-trimoxazole, Ertapenem, Norfloxacin, Piperacillin, Ticarcillin	*gyrA*: S83L	TEM-4	ACT- (5,14)	OXA- (1,224,31)	aadA5	QnrB4	sul1	dfrA17	mphA

### Mobile genetic elements carrying AMR gene cassettes

**Fig. 3:**
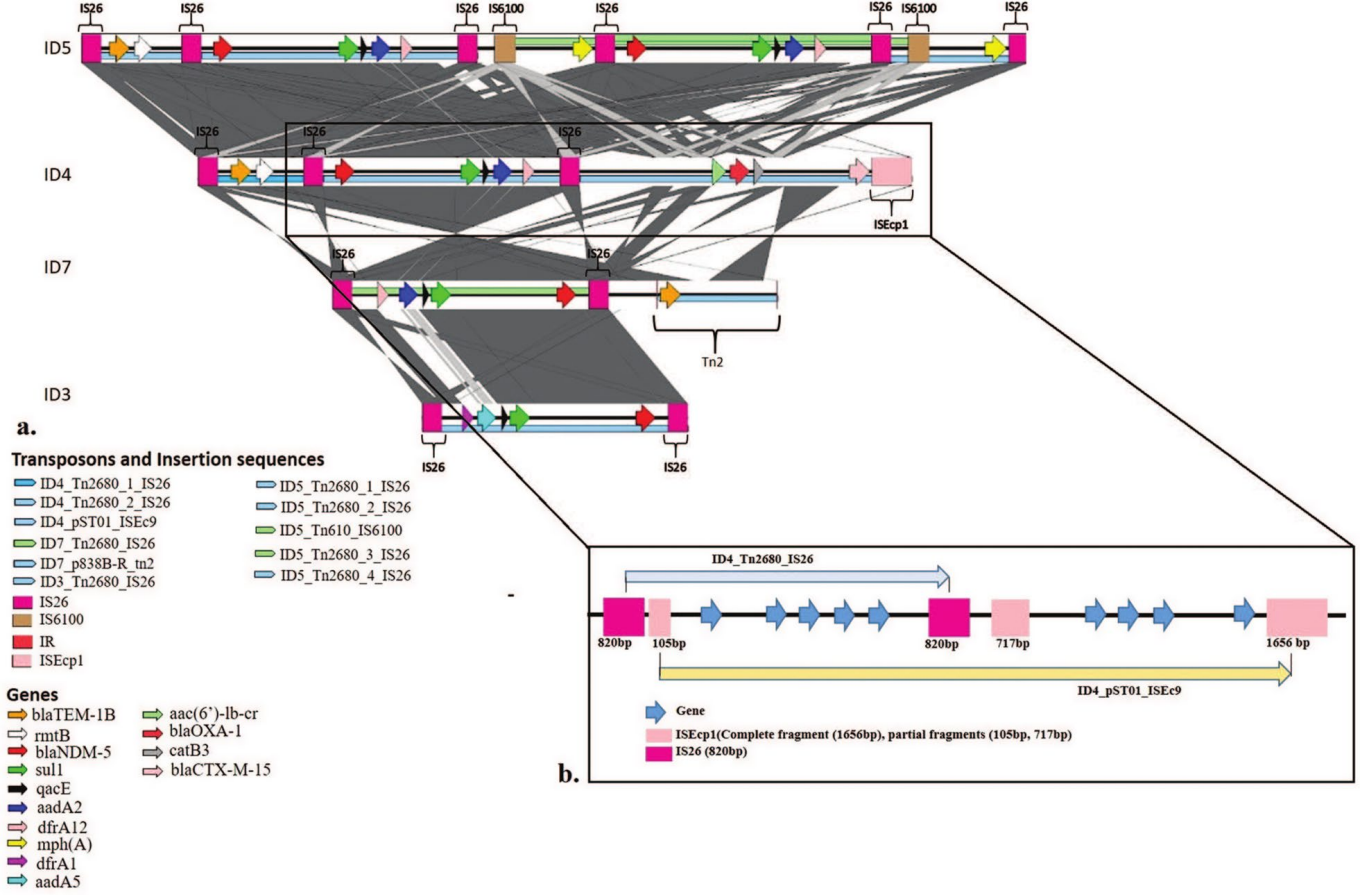
Comparison of mobile genetic elements among the *Escherichia coli* isolates. **a** The figure shows the presence of the mobile elements carrying similar AMR gene cassettes and draws the comparison of these elements between isolates ID5, ID4, ID7 and ID3. All these 4 isolates were seen carrying Tn2680 with the same AMR genes with high similarity to each other bracketed by a set of IS26. The horizontal lines in the background represent the transposons with forward orientation (blue) and reverse orientation (green). The squares represent the insertion sequences that form the transposon and the arrows represent the genes carried by the transposons. The dark gray shaded area indicates the regions with high identity between the isolates. **b** The region inside the box has been zoomed in to show the co-integrated mobile genetic elements. The partial fragments of ISEcp1(105 bp, 717 bp) were seen lying in close proximity to the insertion sequences (IS26) of Tn2680 and surrounding the gene cassette containing *bla*_NDM-5_, aadA2, sul1, dfrA12 and qacE. Other resistance genes such as aac(6ʹ)-lb-cr, *bla*_OXA-1_, catB3 and *bla*_CTX-M15_ with a complete ISEcp1 fragment was found downstream of the 717 bp partial fragment

A composite transposon, Tn2680 was commonly found in seven out of 19 isolates (ID4, ID5, ID6, ID3, ID18, ID2, ID7). The genetic context of this transposon was found to be varying, carrying different AMR genes cassettes. In four isolates (ID7, ID3, ID4, ID5), this transposon was found carrying *bla*_NDM-5_, *aadA2*, *sul1*, *dfrA1*, and *qacE* genes together with IS26 insertion sequence (Fig. 3). It was also found to carry *bla*_TEM-1B_ and *rmtB* genes in two isolates (ID4 and ID5) whereas in two other isolates (ID18 and ID4), it harboured *bla*_OXA-1_, *catB3* and *aac(3)ld* genes. Since this transposon was observed with similar genetic context in several isolates, it has the potential to disseminate the multi-drug resistance in the same or even the other bacterial strains. A 14 kb unit transposon (Tn7) was found harbouring *dfrA1* and *aadA2* genes in ID11. Another 4 kb unit transposon (Tn2) known to have originated from plasmid p838B-R observed carrying *bla*_TEM-1B_ in ID14 isolate.

Two composite transposon carrying virulence genes (*iutA*, *iucC*, *sat*, *papA*, *papC* and *iha* responsible for siderophore, aerobactin, autotransporter toxin) were found in ID3 and ID4 isolates. In ID3, it was associated with insertion sequence IS629 carrying *iutA* and *iucC* genes. In ID4, two transposons with ISEc43 sequences at both ends were found overlapping each other (one IS carrying *iucC*, *iutA* and *sat* gene while the other was carrying *papA_F7-2*, *papC* and *iha* virulence genes). No specific pattern of dissemination of AMR genes in different geographical regions was observed in our study.

### Acquisition of *bla*_NDM-5_, *bla*_OXA_ and *bla*_CTX-M15_ into *E. coli*

*bla*_NDM-5_ was the only variant of *bla*_NDM_ observed to be associated with mobile elements in our study. It was seen to be carried commonly with a composite transposon, Tn2680 in close association with other AMR genes such as *aadA2*, *sul1*, *dfrA1*, *qacE* and *mphA* in few isolates.

Another composite transposon, originally associated with plasmid pST01 belonging to IS1380 family with insertion sequence ISEc9 was found with 100% coverage and 100% identity in isolate ID4. It was seen carrying a similar gene cassette formed with *bla*_NDM-5_, *aadA2*, *sul1*, *dfrA1*, *qacE* carried by Tn2680 in addition to few other genes such as *bla*_OXA-1_, *bla*_NDM-5_, *bla*_CTX-M-15_, *aac(6ʹ)lb-cr* and *catB3*. The end of the transposon was seen with complete insertion sequence of ISEcp1, however, partial insertion sequence fragments of 105 bp and 717 bp of ISEcp1 were found upstream and downstream of gene cassette. Insertion sequence IS26, also was observed to be present in close proximity to the 717 bp ISEcp1 fragment. It seems plausible that two transposons carrying different gene cassettes have been co-integrated together and can be mobilised as one whole mobile genetic element. However, the mobility of the integrated element has to be investigated further.* bla*_OXA-1_ was seen being carried on Tn2680 in two isolates (ID18 and ID4). *bla*_CTX-M15_ was found associated with Tn2680 in two isolates (ID2, ID6) and ISEc9 insertion sequence in isolate 4. Isolate 3 harbored *bla*_CTX-M-15_. However, no association with any mobile element was seen.

## Discussion

The present study focuses on paediatric population reporting the molecular epidemiology and various resistance markers of *E. coli* isolates from various community centers of India. Out of 63 *Enterobacterales*, 86% were identified as *E. coli* followed by 11% *K. pneumoniae*. Girls were most commonly affected than boys with a predominance of age group above 5 years. Studies from Israel offer a unique perspective on ESBL-producing infections in children from the Middle East, where a significant uptrend was found in the yearly incidence of paediatric ESBL-positive UTI infections from 1.2 to 5.2% during the study period 2008 to 2011.The ESBL positivity among *E. coli* in our study was around 63%. Similar findings of *E. coli* isolates have been obtained from other studies conducted in community settings in different geographical regions. [4, 29–31].

Earlier, studies have shown that CTX-M-type ESBLs have replaced TEM- and SHV-type ESBLs in Europe, Canada, and Asia as the most common ESBL type among various members of the Enterobacterales. Additionally, earlier molecular epidemiology studies have reported that one specific *E. coli* clone i.e. ST131 has been highly associated with the production of CTX-M resistance in gram-negative infections in paediatric populations [7].

In the current study, ST131 phylogroup was the most prevalent (15.8%) among the identified sequence types of *E. coli* isolates, which has been shown to be a high-risk clone [32]. More than 66% of the *E. coli* isolates belonging ST131 were carrying *bla*_CTX-M-15_ gene. ST167, another high-risk clone was also identified (10.5%) harboring *bla*_NDM-5_ along with multiple *bla*_NDMs_ [33]. Three of our isolates (15.8%) could not be assigned to any known sequence type based on the seven-gene Achtman scheme described at https://pubmlst.org/ [34]. In the present study, IncF plasmids were known to carry ESBL genes especially *bla*_NDM_, genes for aminoglycoside modifying enzyme and plasmid-mediated quinolone resistance (PMQR) [35]. The emergence of a variety of CTX-M-positive *E. coli* isolates in paediatric populations poses a serious threat, as beta-lactams are often the first line of therapy for UTIs and fluoroquinolones are not routinely used in these populations. The increasing prevalence of CTX-M-15-harboring *E. coli* ST131 strains in children as demonstrated by this study has important clinical and public health implications due to the risk of treatment failure. Particular to community-acquired ESBLs, UTI rates ranged from 3.8% to as high as 43% in European children with multiple genitourinary comorbidities [31]. European studies on the molecular epidemiology of ESBL-producing isolates in children are primarily small, single-center cohort studies. However, overall trends are consistent with the global shift to CTX-M–type ESBL dominance [31]. Another report from French children highlights 44% of community-acquired ESBL positive clinical isolates harbouring *bla*_CTX-M-15_followed by*bla*_CTX-M-14_. More than 60% of isolates of the B2 phylogroup strains in both healthcare and community-acquired infections belonged to the ST131 clone [31].

A study in 2017 from South India investigated the prevalence of ESBL levels and associated genes present in *E. coli* from paediatric UTI [36]. The prevalence of ESBL producer was observed as 37.5% in which *bla*_CTX‑M_ gene being the most prevalent (87.5%), followed by *bla*_TEM_ (68.4%) and *bla*_SHV_ (3.1%). 63% (35/54) ESBL positivity was observed in the current study among the circulating *E. coli* isolates of which *bla*_CTX-M_ (66.6%; 10/15) as the most prevalent followed by *bla*_TEM_ (60%; 9/15).

In the present study, various plasmid-mediated AMR genes along with chromosomal point mutations and virulence genes were found in the *E. coli* isolates. The presence of high-risk clones in these isolates from paediatric patients suffering with UTI in the community setting of India is worrisome.

## Conclusions

The incidence of antibiotic resistance in the community-onset UTIs caused by ESBL-producing carrying high-risk resistant clones of *E. coli* among children seems to be increasing in India; thereby management of UTI in paediatric patients might be challenging for the clinicians in near future. This study demonstrates a need for heightened awareness regarding the increasing frequency of these resistant isolates in the paediatric population in the community and their potential impact on disease management. Rapid detection with One health approach is the need of the hour for proper management and prevention of the spread of drug-resistant pathogens in the community. Regular surveillance at different community settings will be helpful to understand the genotypes, their transmission dynamics and further implementation of preventive measures.

## Data Availability

The raw sequencing data reported in this study has been deposited in the European Nucleotide Archive (ENA) under the BioProject Accession Number: PRJEB57435.

## Abbreviations

UTI: Urinary tract infection
UPEC: Uropathogenic *Escherichia coli*
ESBL: Extended-spectrum beta-lactamases
ST: Sequence type
MIC_50_: Minimum inhibitory concentration of 50 percentile
MIC_90_: Minimum inhibitory concentration of 90 percentile
MLST: Multi locus sequence types
ARG: Antibiotic resistance gene
SNP: Single nucleotide polymorphism
AMR: Antimicrobial resistance
MDR: Multidrug resistant
CHC: Community health centers
CFU: Colony forming unit
OPD: Outpatient department
MALDI-TOF MS: Matrix-assisted laser desorption ionization time of flight mass spectrometry
AST: Antibiotic susceptibility testing
WGS: Whole genome sequencing
